# Dermoscopy of Fixed Cutaneous Sporotrichosis With a Follow-Up Till Cure: A Case Report

**DOI:** 10.7759/cureus.55960

**Published:** 2024-03-11

**Authors:** Rohit Kothari, Jeenu Varghese, Anuj Bhatnagar, Karthi Kishore, Rahul Kumar

**Affiliations:** 1 Dermatology, Command Hospital Air Force, Bangalore, IND; 2 Pathology, Command Hospital Air Force, Bangalore, IND

**Keywords:** amphotericin-b, cure, dermoscopy image analysis, cutaneous sporotrichosis, literature review of disease

## Abstract

Fixed cutaneous sporotrichosis (FCS) is a rare and chronic infection. Its diagnosis requires a high degree of suspicion. The data on its dermoscopy and follow-up is limited in the literature. We herein report one such case with a follow-up till cure along with its dermoscopy to establish certain specific features that may be used to ascertain the response to treatment for this chronic infection and its prognosis. We found only three such cases following an extensive review of the literature, and this case emphasizes the importance of dermoscopy in recent times as the history, swab cultures, and smears may be misleading at times due to the chronic and long-standing nature of the condition.

## Introduction

Fixed cutaneous sporotrichosis (FCS) is a rare presentation of sporotrichosis that remains localized at the inoculation site instead of the usual linear spread along the lymphatics. There is not enough data on its dermoscopy [1]. Till date, only three cases of dermoscopy of FCS have been described, none with a dermoscopy follow-up to establish the specific features that may be used to ascertain response to the treatment in this chronic infection and its prognosis [2,3]. We herein present one such case with a dermoscopy follow-up and a thorough review of the literature.

## Case presentation

**Figure 1:**
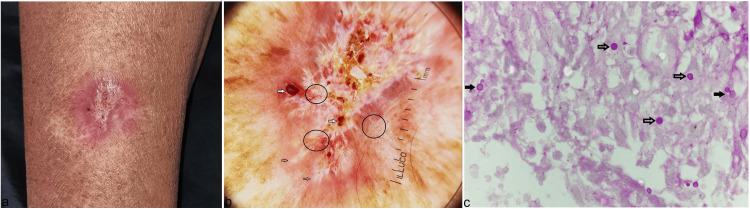
A solitary erythematous, crusted, indurated, and ulcerated plaque over the dorsum of the right forearm (a). Dermoscopy (Illuco IDS-110, Polarized 10x) showing a reddish-orange background with erythema, white structureless areas with leaf-venation-like appearance at the peripheries (black empty arrows), white scales, predominantly peripheral polymorphic vessels (coiled, loop, dotted, and curved) (black circles), yellowish crust and superficial ulcerations in the center, and hemorrhages (white solid arrows) (b). Histopathology of the lesion showing numerous budding (black solid arrows) and non-budding (black empty arrows) periodic acid-Schiff positive yeast forms measuring 5-7 micrometers with narrow base in the dermis (H&E, 1000x) (c).

A 52-year-old immunocompetent female presented with a solitary non-tender erythematous crusted, indurated, and ulcerated plaque over the dorsum of her right forearm for the last three years, with occasional seropurulent discharge and pruritus (Figure 1a). She denied any trauma, contact with animals, gardening, or skin disease before the lesion. She had received multiple courses of oral/topical antibiotics, steroids, and antifungals intermittently without any relief. Dermoscopy of the lesion revealed an orange-yellow background with erythema, white structureless areas with a leaf-venation-like appearance at the peripheries, white scales, predominantly peripheral polymorphic vessels (coiled, loop, dotted, curved), yellowish crust, and superficial ulcerations in the center, and hemorrhages (Figure 1b). There was no lymphadenopathy, and the systemic examination was normal. Swab cultures from the lesion for bacteria, fungus, *Mycobacterium tuberculosis*, and atypical mycobacterium were negative, and swab smears for Ziehl-Neelsen (ZN) and gram stain were also negative. The X-ray of the right forearm was normal. A skin biopsy taken from the lesion with differentials of squamous cell carcinoma, discoid lupus erythematosus, atypical mycobacterial infection, deep fungal infection (fixed cutaneous sporotrichosis), and cutaneous leishmaniasis revealed thinned out stratified squamous epithelium with parakeratosis and focal areas of ulceration with underlying dermis showing dense inflammatory infiltrate comprising of neutrophils, plasma cells, eosinophils, and lymphohistiocytoses. There were numerous budding and non-budding periodic acid-Schiff positive yeast forms, measuring 5-7 micrometers with a narrow base in the dermis and within the polymorphs (Figure 1c).

**Figure 2:**
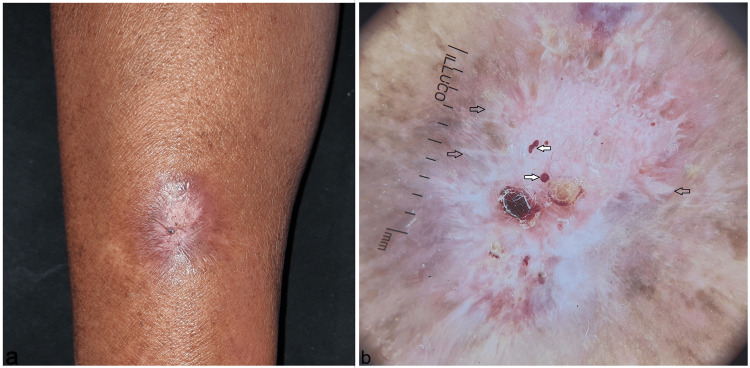
Follow-up at eight weeks showed a significantly healing lesion with minimal erythema and healed ulcerations (a). Dermoscopy (Illuco IDS-110, Polarized 10x) showing a marked reduction in erythema, reddish-orange background, polymorphic vessels (black circles), hemorrhage (white solid arrows), crusting, and superficial ulcerations; and prominent white structureless areas with a leaf-venation-like appearance at the peripheries (black empty arrows) and scaling (b).

Tissue polymerase chain reaction (PCR) for *Mycobacterium tuberculosis* was negative. Tissue culture on Sabouraud's dextrose agar grew cream-colored moist colonies of Sporothrix species. She was diagnosed with fixed cutaneous sporotrichosis and was treated with oral itraconazole 200 mg daily and topical 0.1% liposomal amphotericin-B cream twice daily for three months. Follow-up at eight weeks showed a lesion showing significant healing (Figure 2a) and the dermoscopy revealed a marked reduction in erythema, reddish-orange background, polymorphic vessels, hemorrhage, crusting, and superficial ulcerations, signifying resolution of the inflammation and dermal granulomas; however, there were prominent white structureless areas with leaf-venation like appearance at the peripheries and scaling, signifying dermal scarring in a resolving lesion and an adequate response to the treatment (Figure 2b).

**Figure 3:**
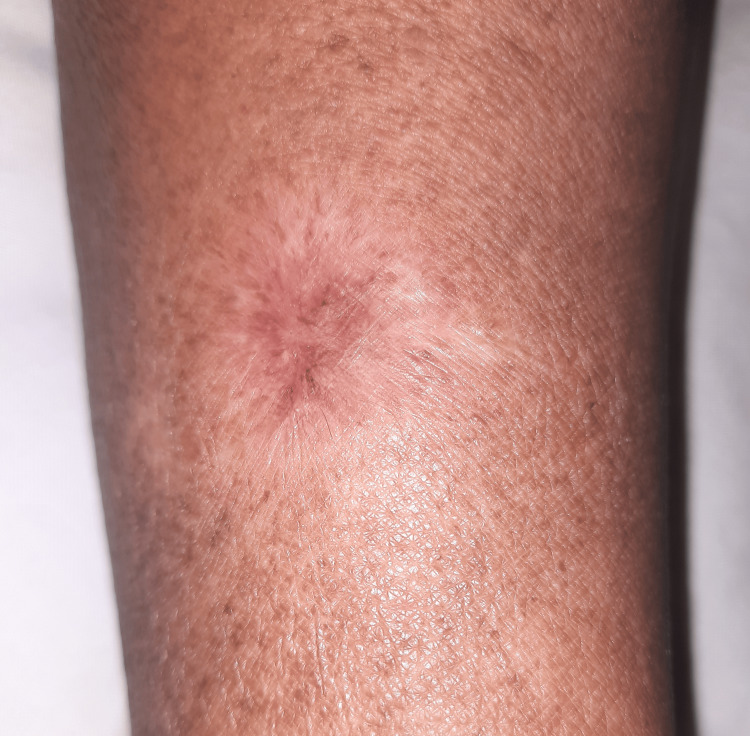
Follow-up at three months revealed complete resolution with scarring of the lesion.

Follow-up at three months revealed complete resolution of the lesion with scarring (Figure 3).

## Discussion

Sporotrichosis is an uncommon chronic subcutaneous granulomatous infection caused by the saprophytic fungi *Sporothrix schenckii*. The lymphocutaneous variant is the most common, accounting for ~70-80% of cases [4]. Sporothrix is a common saprophyte of plant detritus, soil, and moss, and the infection is endemic in the tropics and subtropics. The professions that are more predisposed to developing this condition include gardeners, agriculturists, florists, foresters, and people handling plants or plant material. The most common route for acquiring this infection is through traumatic inoculation.

Fixed cutaneous sporotrichosis is a rare variant that presents as a localized asymptomatic erythematous papule, pustule, plaque, or nodule and may progress to a non-healing ulcer or localized abscess at the inoculation site [5]. Tissue culture is considered the gold standard diagnostic modality. Demonstration of budding forms of the yeast that may be cigar-shaped is diagnostic; however, it is often not seen due to its scanty presence [6]. Itraconazole is considered the drug of choice for mild to moderate cases of cutaneous sporotrichosis as per the published practice guidelines [7]. There is limited data on its dermoscopy in the literature. The salient features that have been described and were also seen in our patient were an orange-yellow background with erythema corresponding to the dermal granuloma with inflammation, hemorrhages, yellowish crusts, white structureless areas showing dermal scarring, superficial ulcerations, scaling, and polymorphic vessels. Yellow tears have been described specifically for the facial lesions and correspond to follicular plugs due to compression of follicular openings from the lesional growth [2].

## Conclusions

To conclude, more data on dermoscopy of FCS may aid in its diagnosis, prognosis, and establishing the response to the treatment. The history, swab cultures, and swab smears may be misleading at times, and dermoscopy may be considered an important tool in the management of this chronic and rare condition.
